# Journals accepting case reports

**DOI:** 10.5195/jmla.2023.1747

**Published:** 2023-10-02

**Authors:** Terri Gotschall, Angela Spencer, Margaret A. Hoogland, Elisa Cortez, Elizabeth Irish

**Affiliations:** 1 terri.gotschall@ucf.edu, Scholarly Communications Librarian, Health Sciences Library, University of Central Florida College of Medicine, Orlando, FL.; 2 angela.spencer@slu.edu, Saint Louis University, St. Louis, MO.; 3 Margaret.Hoogland@Utoledo.edu, Associate Professor, University Libraries, The University of Toledo, Toledo, OH.; 4 elisa.cortez@ucr.edu, Medical Education and Clinical Outreach Librarian, UC Riverside Library, Orbach Science Library, University of California, Riverside, Riverside, CA.; 5 irishe@amc.edu, Associate Professor, Schaffer Library of Health Sciences, Albany Medical College, Albany, NY.

**Keywords:** Case Reports, Journals, Publishing

## Abstract

**Background:**

Few resources exist to support finding journals that accept case reports by specialty. In 2016, Katherine Akers compiled a list of 160 journals that accepted case reports, which many librarians continue to use 7 years later. Because journals' editorial policies and submission guidelines evolve, finding publication venues for case reports poses a dynamic problem, consisting of reviewing a journal's author guidelines to determine if the journal accepts case report manuscripts. This project aimed to create a more up to date and extensive list of journals that currently accept case reports.

**Case Presentation:**

1,874 journal titles were downloaded from PubMed. The team reviewed each journal and identified journal titles that accept case reports. Additional inclusion factors included being indexed in MEDLINE, accessible on the internet, and accepting and publishing English language submissions.

**Discussion:**

The new journal list includes 1,028 journals covering 129 specialties and is available on the Open Science Framework public page.

## BACKGROUND

Case reports are a type of article that provide a detailed account of a novel clinical finding, describing the findings, clinical progress, and future outlook of an individual patient [1]. Though the gold standard in publishing is original research, publishing a case report continues to add value to the medical literature [2]. A case report may also serve as a starting point for more complicated studies and research projects [3]. However, peer-reviewed journals in the last twenty years have become more reluctant to accept case reports as they are low on the hierarchy of evidence [4]. One example of this is the American College of Cardiology journal, which directs case reports to the Journal of the American College of Cardiology (JACC) Case Reports publication, rather than other JACC titles. JACC views case reports as an opportunity for early career cardiologists to start their publication journey but expects more experienced practitioners to publish their research within other formats [5].

The design of contemporary undergraduate medical education has reinforced the importance of case report authorship as a publishing opportunity for early career physicians. Fourth-year medical students have expressed that writing a case report improves their scientific writing and presentation skills, improves their curriculum vitae, and helps them secure residency positions [4]. The pressure on medical students to publish has increased after the transition of the United States Medical Licensing Examination (USMLE) Step 1 from a graded score to pass/fail in January 2022. This change may be one of the driving forces behind medical students seeking more opportunities to publish, as students find ways to make their residency applications stronger [6]. While the impact of the new USMLE scoring on student ranking in the National Resident Matching Program (NRMP) is too early to measure, initial results from program director surveys indicate that research will become a more important metric in residency selection [7-9].

Despite the availability of articles providing guidance on how to write case reports and select journals, authors are still faced with the challenge of finding a journal in their specialty that accepts case reports [10-13]. Consequently, librarians and informationists often receive requests from authors seeking assistance in locating journals that accept case reports. To help librarians, in 2016, Akers compiled a list of 160 journal titles that accept case reports [14]. However, the number of journals that accept case reports has grown over time. To address this need, our team aimed to create an extensive, but not exhaustive, list of journals that currently accept case reports, and make it publicly available. This list can serve as a valuable resource for authors, serving as a starting point for finding journals in their specialty, while also offering the opportunity for librarians and informationists to customize it to meet the needs of their users.

## CASE PRESENTATION

### Journal Search

Our team conducted a search in PubMed for journals that included the publication type “case reports.” Next, the filters for “MEDLINE” and “English” were applied. Lastly, the team used the custom date ranges from November 1, 2021, to April 30, 2022, to narrow down to journals that were currently publishing. The records were downloaded to Excel and journal titles were deduplicated.

The team chose to limit to MEDLINE journals. Indexing requirements for MEDLINE include transparent editorial policies, explicit peer review information, ethical and conflict of interest policies, and editorial board information [15]. Additionally, it is possible to search MEDLINE-using a variety of databases and search engines, which makes articles published in MEDLINE indexed journals discoverable to a wider range of potential authors who may have different access points to MEDLINE.

### Journal Evaluation and Data Collection

The team collected the following information for each journal: journal title, URL to instructions to authors, and whether the journal accepts case reports. For any journal currently accepting case reports, the team collected further information about the publishing model (subscription with no author fees, subscription with author fees, open access with no fees, open access with fees, or hybrid) and selected the appropriate specialties. To facilitate data collection, the National Library of Medicine (NLM) catalog was searched for each journal, and from the journal record the electronic links were used to navigate to the journal websites. If a link was broken the team searched the internet for the journal title.

The team created a controlled vocabulary list for the specialties. For cross-disciplinary journals, the team selected all applicable specialties. For journals with unclear specialties, the team included all the assigned Medical Subject Headings (MeSH) terms listed in the NLM catalog record. Team members selected “other,” when a specialty did not appear on the list, then reviewed and updated the list as needed. Post-screening, the team finalized the specialty list.

Finally, the team used the free version of AirTable (Formagrid, Inc., San Francisco, CA), which includes an online data collection form. After compiling the data, the team uploaded the file into the Open Science Framework.

### Results

Of the 1,874 journals reviewed, the team excluded 846 titles for the following reasons:

1. Not fully indexed by MEDLINE (n=3)
2. The journal website or instructions to authors used non-English text and the team could not translate the instructions into English (n=30)
3. Unable to access the author instructions or broken journal websites (n=5)
4. The journal was no longer being published (n=2)
5. Non-journal publications (conference proceedings and books) (n=3)
6. Did not explicitly state in the author guidelines that accept case reports (n=803)

The final list includes 1,028 journals and is available in the Open Science Framework (OSF) at https://osf.io/b9wnx. Users can download the list or view it online. The spreadsheet is searchable, and users can sort the list by clicking on the column header(s).

## DISCUSSION

Authors often consult librarians about where to publish articles. During a consultation or reference interview, the librarian may guide authors through finding and evaluating journals. They may help authors create their own list of criteria for a journal including manuscript types accepted, indexing, aim and scope, open access, cost to publish, or journal ranking [16]. Once an author has a list of criteria, a librarian or author will need to find journals to submit their article to, this is often a time-consuming process. Two common criteria for authors of case reports are the journal must accept case reports and the journal is indexed in MEDLINE. Having a list of journals by specialty, indexed in MEDLINE, and accepting case reports is a useful starting point for librarians and authors.

Case reports are not usually funded research projects and some authors may have to consider the cost of publishing their manuscript. Costs could vary by geographic location or the journal's agreement with an author's organization [17, 18], though some authors have access to departmental funds to cover publishing costs. For librarians at organizations that do not provide funds, authors frequently ask for journals that are 100% free of Article Processing Charges (APCs), submission fees, page charges, and color fees. This usually leads librarians to discuss the benefits and challenges of various publishing models. By including the journal's publishing model, broken out to include open access with and without APCs, and subscription journals with and without fees, authors and librarians can quickly weed out journals by costs. Additionally, authors who want to publish their work open access can quickly determine if a journal offers a route to open access publishing.

The team intends for the list of journals available on Open Science Framework (OSF) to be customized for local use. The list can be downloaded and further curated to meet a specific user group's needs, for example by adding journals that are not indexed in MEDLINE, or by showcasing open access journals with transformative agreements with the organization. The curated list can then be uploaded and shared with a user group on the library's website.

## LIMITATIONS

Journals frequently change publishing models, fee structures, and submission categories. As a result, any listing of journals that publish case reports represents at best a partial snapshot of the landscape at a given time. While this list can serve as a starting point when consulting with learners about where to publish a case study, it should not be viewed as exhaustive and may become out of date.

## Data Availability

Medical Journals that Accept Case Reports is available on Open Science Framework (OSF) under the CC-By Attribution 4.0 International Creative Commons license at https://osf.io/b9wnx.
